# Changes in shoulder outcomes using ultrasonographic assessment of breast cancer survivors: a prospective longitudinal study with 6-month follow-up

**DOI:** 10.1038/s41598-021-02379-9

**Published:** 2021-11-26

**Authors:** Paula Gala-Alarcón, Virginia Prieto-Gómez, Javier Bailón-Cerezo, María José Yuste-Sánchez, Beatriz Arranz-Martín, María Torres-Lacomba

**Affiliations:** Physiotherapy in Women’s Health (FPSM) Research Group, Physiotherapy Department, Faculty of Medicine and Health Sciences, University of Alcalá, 28805 Madrid, Spain

**Keywords:** Breast cancer, Pain, Musculoskeletal system

## Abstract

This study aimed to describe changes in supraspinatus tendon thickness, acromiohumeral distance, and the presence of fluid in the subacromial bursa as measured by ultrasound, as well as shoulder range of motion and strength, perceived shoulder disability, and health-related quality of life in women before and after breast cancer treatment. Women who underwent surgery for unilateral breast cancer who did not suffer from shoulder pain or difficulty performing activities of daily living in the 6 months prior to surgery were included. One pre-surgical (A_0_) and three post-surgical assessments at 7–10 days (A_1_), 3 months (A_2_), and 6 months (A_3_) after surgery were carried out. The thickness of the supraspinatus tendon on the affected side decreased between post-surgical (A_1_) and 6-month (A_3_) follow-up assessments (p = 0.029), although the minimal detectable change was not reached. The active range of motion of the affected shoulder decreased after surgery. Strength changes were observed in both shoulders after surgery**. **The intensity of shoulder pain increased between post-surgical and 6-month follow-up assessments. Shoulder function was decreased at the post-surgical assessment and increased throughout the follow-ups. Health-related quality of life declined after surgery. A trend of decreasing thickness of the supraspinatus tendon of the affected shoulder was observed. Detecting these possible structural changes early would allow for early or preventive treatment.

## Introduction

Breast cancer is the second most prevalent cancer in the world after lung cancer, and it is the most frequent cancer in women. In 2012, 1.67 million new cases of breast cancer were identified worldwide, accounting for 25% of all cancers. Its incidence rate is higher in developed countries^1^. Medical treatments for breast cancer lead to sequelae that can affect the upper limb, even in the long term. Shoulder and arm pain decreased shoulder range of motion, stiffness of the major and minor pectoral muscles, decreased strength and activity of the shoulder and scapular muscles, and lymphedema are the main shoulder-related sequelae after medical treatments^2^. All of these sequelae have a negative impact on the quality of life of breast cancer survivors, especially with regard to body changes, negative self-evaluation, and concerns about cancer^3^. Surgery and other medical treatments can damage neuromuscular and vascular tissues^4–7^, and radiation therapy and chemotherapy, as well as lymphadenectomy and mastectomy, appear to be related to more severe shoulder pain and increased shoulder mobility restriction^8–12^. Most current studies suggest that the peripheral sensitization that may be present in shoulder pain is likely related to inflammatory processes associated with tissue lesions^13^. This nociception generates changes in shoulder motor strategies, which in turn can overload peripheral structures^14^. Therefore, the aftermath effects of breast cancer treatment on the shoulder may be linked to the development of pathology in the rotator cuff of the affected side^6^. The decreased supraspinatus tendon thickness in overloaded or wheelchair-bound athletes^15,16^ is thought to be due to the tendon's response to traction force, including the intratendinous fluid moving away from it. In this way, sustained overload on the supraspinatus tendon due to pain-related disuse muscle atrophy could result in some reduction in the thickness of the tendon in breast cancer survivors.

Ultrasound (US) provides an objective, dynamic, fast, effective, safe, real-time, and comparative study of musculoskeletal tissue. US assessment allows direct visualization of the structure and function of muscles, tendons, ligaments, and nerves to relate them to clinical symptoms. US also facilitates decision-making in physiotherapy assessment and diagnosis, treatment planning, intervention, and reassessment in patients with pain and/or dysfunction in body regions such as the low back, knee, or shoulder^17^. This study aimed to describe changes in supraspinatus tendon thickness, acromiohumeral distance, and the presence of fluid in the subacromial bursa as measured by US; changes in the range of motion and strength of the shoulder on the affected and contralateral sides; and changes in the self-reported functional status throughout different physiotherapy assessments: pre-surgical and 7–10 days, 3 months, and 6 months after surgery.

## Methods

We carried out a prospective longitudinal study between November 2018 and June 2020 at the Physiotherapy in Women’s Health (FPSM) Research Unit of the Alcalá University (Madrid, Spain). This study was approved by Clinical Research Ethics Committee of the Principe de Asturias Hospital (HUPA) in Alcalá de Henares (Madrid, Spain) (OE 24/2018).

Women diagnosed with unilateral breast cancer without shoulder pain or difficulty in performing activities of daily living 6 months prior to breast surgery were recruited from HUPA by the medical staff, and referred to FPSM for a pre-surgical physiotherapy assessment to verify that they met the selection criteria. Eligible women gave written informed consent to participate in the study after breast cancer had been confirmed by biopsy. We excluded women with neurological problems such as decreased motor power, sensory changes, and decreased deep tendon reflexes, upper limb surgery, and/or cognitive problems that prevented the completion of questionnaires. Each participant was assessed preoperatively (3–5 days before surgery (A_0_)). The main outcome was supraspinatus tendon thickness.

### Follow-up

Initially we scheduled 3 follow-up visits: at 7–10 days (A_1_) and 3 and 6 months (A_2_, A_3_) after surgery. These dates were flexible, depending on the participant’s availability.

All women received information on the side effects of surgery (seroma, axillary web syndrome (AWS), impaired shoulder movement, impaired arm sensitivity, pain); the anatomy, physiology, and physiopathology of the lymphatic and venous systems; concepts of normal load versus overload; causes of AWS and secondary lymphedema; identifying possible precipitating factors; and the four categories of interventions to prevent secondary lymphedema (prevention of infection, avoidance of arm constriction, and use of the arm). Regarding the use of the arm, all women received the same information at hospital discharge, i.e., that they move the arm as they normally did in their activities of daily living, progressing as post-surgical pain permitted.

If women experienced pain, AWS, or any other symptoms, they could contact the physiotherapist and a visit would be arranged^18^. If secondary lymphedema was diagnosed, then complex decongestive physiotherapy was carried out^19^.

### Assessment

A trained physiotherapist with at least 5 years of experience in musculoskeletal US was responsible for the physical and US assessment and for entering outcome data on a data sheet. At all visits, the same US protocol and physical examination of the shoulder were performed by the same physiotherapist. This physiotherapist performed the pre-surgical assessment and all 3 post-surgery follow-up assessments of all women.

#### Thickness of the supraspinatus tendon

Data were collected by US scanning. The intra-examination minimum detectable change (MDC) for a single examiner is 0.6 mm^20^. The acromiohumeral distance and the presence of fluid in the subacromial bursa (> 2 mm) were also collected^20,21^. The US protocol used to measure these outcomes can be found in Supplementary File S1. To control for US measurement biases, 2 measurements were taken, and the mean was used. Similarly, the US assessment protocol was always performed first on the right shoulder and then on the left, regardless of which side was affected.

#### Pain

The presence of pain disorder with neuropathic characteristics was measured using the LANSS scale, which is a 7-item self-administered scale with dichotomous responses. The total score ranges from 0 to 24 points, and a score of ≥ 12 indicates neuropathic pain^22^. The intensity of shoulder pain was measured with a visual analogue scale (VAS). The MDC in people with pain related to rotator cuff disease is 14 mm^23^.

#### Shoulder active range of motion and muscle strength

Active glenohumeral flexion, extension, abduction, adduction, and medial and lateral rotation range of motion of both shoulders were measured in degrees using a digital inclinometer (Baseline®, Fabrication Enterprises Inc., New York, NY, USA) with the participant in a sitting position with feet supported, knees flexed at 90°, and a straight back. The MDC in the breast cancer population is 20° for abduction, 10.2° for flexion, 13.6° for extension, 9.2° for internal rotation, and 20.1° for external rotation^24^. In the same position, submaximal (as a precaution after surgery) isometric contraction strength in flexion, extension, abduction, and adduction movement and medial and lateral rotation of the shoulders was measured in newtons using a dynamometer (MicroFET®2, Hoggan Health Industries, West Jordan, UT, USA) placed in the distal third of the humerus without including the elbow joint. Two contractions of 3 s were requested, with 5 s of rest, and the mean was registered. Submaximal isometric contraction measured with a dynamometer has shown good reliability^24^. The MDC in healthy people is a strength change of more than 15% in any position.

#### Perceived shoulder pain and disability

These were measured with the Spanish version of the Shoulder Pain and Disability Index Questionnaire (SPADI), which consists of 13 items divided into two subscales: pain (5 items) and disability (8 items). Scores on both subscales are rated from 0 (no pain or disability) to 10 (worst pain imaginable or so difficult to perform tasks that help is required). The higher the score on each subscale, the greater the intensity of pain and disability. The SPADI has proven to be a reliable, valid, and sensitive instrument to assess shoulder symptoms and quality of life in Spanish women after breast cancer treatment. The MDC in this population is 17 points in relation to the total score^25^.

#### Health-related quality of life (HRQoL)

This was assessed using the Spanish version of the Functional Assessment of Cancer Therapy–Breast Questionnaire 4 (FACT-B + 4). It consists of 41 items and various domains, including the FACT-General (FACT-G) and specific scales for breast and upper limb cancer (BCS). The FACT-G measures 4 dimensions of well-being: physical (PWB), emotional, social, and functional (FWB). Three scores are obtained: the FACT-G score as a sum of the 4 generic dimensions, the FACT-B score as the total for the BCS scale, and the Trial Outcome Index (TOI) score as the sum of the PWB, FWB, and BCS scales. Higher scores indicate better quality of life. The MDC is defined as 5 points for the FACT-G, 2 points for the BCS scale, 7 points for the FACT-B, and 5 points for the TOI^26^.

#### Other variables

During the preoperative assessment, we collected personal data on the participants, including age, body mass index, menopause status, previous pathology of contralateral shoulder, and medical history. In postoperative assessments, data were collected on the type of breast and axillary surgery done and the use of adjuvant treatment (chemotherapy, radiotherapy, and/or hormone therapy).

### Sample size estimation

The a priori sample size estimation was calculated for 2 repeated paired samples in a group. When accepting an alpha risk of 0.05 and a beta risk of 0.2 in a bilateral contrast, 35 subjects are needed to detect a difference equal to or greater than 1 mm. A standard deviation of 2 mm was assumed based on a previous study^21^, with the expectation that it would increase due to possible variability in the data. Possible loss to follow-up of up to 10% was estimated. Sample-size estimation was made using the statistical program EPIDAT 3.1 (Xunta de Galicia, Spain, 2006).

### Statistical analysis

SPSS Statistics software (version 18, IBM) was used for data analysis. For continuous quantitative variables, the mean and standard deviation were used. Scores on the FACT-B + 4 and SPADI questionnaires were treated as continuous quantitative variables and expressed as the median and interquartile range. Categorical variables were described with absolute values and frequencies. The Shapiro–Wilk statistical test and histogram graphs were used to determine the normality of the data. Student’s t-test was used to estimate the average change between baseline (A_0_) and subsequent assessments (A_1_, A_2_, and A_3_) for each US outcome. The Friedman test was used for the range of motion, strength, health-related quality of life, and perceived shoulder disability outcomes. We used the Pearson correlation coefficient and scatterplots to analyze the association of quantitative variables. In all cases, a p-value of < 0.05 was used. Effect size for repeated measures (σD = σ·2·(1-ρ))^27^ was calculated for the main variable when significant differences were detected, considering 0.20–0.49, 0.50–0.79, and > 0.80 to be small, medium, and large effect sizes, respectively^28^.

### Ethical aspects

All procedures performed in studies involving human participants were in accordance with the ethical standards of the Human Research Ethics Committee at the University of Alcalá, Madrid, Spain (protocol number: OE 24/2018) in Alcalá de Henares (Madrid), Spain and with the 1964 Helsinki declaration and its later amendments or comparable ethical standards.

### Consent to participate

All participants provided written, informed consent.

### Consent for publication

All participants provided consent for publication.

## Results

Of the 35 participants who were included, 30 participants finished the study. There were five dropouts in follow-up (two attended only A_0_ (before surgery) and three attended only A_0_ and A_1_ (after surgery) due to the COVID-19 pandemic). So, 30 women were fully monitored, and their data were analyzed.

One woman developed lymphedema at follow-up, and nine women reported AWS.

Table 1 presents the baseline demographics and clinical pre-surgical descriptive statistics. Baseline measurements showed no significant differences (p > 0.05) in supraspinatus tendon thickness, acromiohumeral distance (AHD), range of motion, or muscular strength between both shoulders.

**Table 1:** Participant characteristics.

**Sociodemographic and anthropometric data (N = 30)**	
Age (years, $\overline{X}$ ± SD)	53.6 ± 9.3
BMI (Kg/m^2^, $\overline{X}$ ± SD)	26.5 ± 6
Menopause (n (%))	19 (63.3)
**Clinical data**	
Previous pathology of contralateral shoulder (n (%))	7 (23.3)
**Axillary surgery**	
Lymphadenectomy (n (%))	9 (30)
Sentinel lymph node biopsy (n (%))	21 (70)
**Breast surgery**	
Mastectomy (n (%))	4 (13.3)
Mastectomy with breast reconstruction (n (%))	13 (43.3)
Lumpectomy (n (%))	13 (43.3)
**Medical adjuvant treatments**	
Chemotherapy pre-surgical (n (%))	6 (20)
Chemotherapy post-surgical (n (%))	17 (56.6)
Radiotherapy (n (%))	21 (70)
Hormone therapy (n (%))	22 (73.3)

*N* Sample size, $\overline{X}$ mean, *SD* standard deviation, *BMI* body mass index, *Kg* kilograms, *n* number of cases.

The number of cases of shoulder pain increased from three to eight between the post-surgical assessment and the 6-month follow-up.

Two women whose axillary surgery was sentinel lymph node biopsy showed neuropathic signs and symptoms according to the LANSS scale in the post-surgical assessment. One of them developed lymphedema, and the other had a wound infection. Two other women with neuropathic pain (> 12 points on the LANSS scale) were identified (lymphadenectomy in both cases).

### Supraspinatus tendon thickness, acromiohumeral distance, and the presence of fluid in the subacromial bursa

When comparing the thickness of the supraspinatus tendon on the affected side between the post-surgical (A_1_) assessment and the 6-month follow-up (A_3_), statistically significant differences (p = 0.029) were found. The mean thickness of the supraspinatus tendon changed from 4.74 ± 1.04 mm to 4.52 ± 0.99 mm, representing a small effect size (*d* = 0.396) that does not reach the MDC^20^. For the other US variables, no differences (p > 0.05) were found (Table 2).

**Table 2:** Shoulder outcomes between baseline measurement and different follow-ups.

Outcomes	Assessments				P-values		
	Pre-surgical A_0_	7–10 days post-surgical A_1_	3 months post-surgical A_2_	6 months post-surgical A_3_	A_0 VS_ A_1_	A_1 VS_ A_2_	A_1 VS_ A_3_
**Affected side**							
Supraspinatus thickness (mm) ($\overline{X}$ ± SD)	4.66 ± 0.94	4.74 ± 1.04	4.55 ± 0.98	4.52 ± 0.99	0.72	0.28	0.03
AHD (mm) ($\overline{X}$ ± SD)	12.45 ± 2.77	12.22 ± 1.77	12.46 ± 2.06	12.38 ± 2.26	0.59	0.24	0.65
**Shoulder active range of motion**							
Flexion (º) Median ± IQR	170.50 ± 37	124.50 ± 80	166 ± 47	172.50 ± 67	< 0.001	< 0.001	< 0.001
Extension (º) Median ± IQR	70 ± 46	60 ± 50	70 ± 30	70 ± 37	0.07	< 0.001	0.001
Abduction (º) Median ± IQR	180 ± 52	110 ± 90	177.50 ± 56	180 ± 70	< 0.001	< 0.001	< 0.001
Aduction (º) Median ± IQR	35 ± 5	35 ± 10	35 ± 5	35 ± 5	0.37	0.34	0.22
External rotation (º) Median ± IQR	107 ± 40	101 ± 46	105.50 ± 36	105 ± 42	> 0.999	0.27	0.41
Internal rotation (º) Median ± IQR	42.50 ± 29	37 ± 42	40 ± 36	36.50 ± 41	0.66	0.50	> 0.999
**Shoulder muscular strength**							
Flexion (N) Median ± IQR	71.55 ± 77.90	47.75 ± 73.90	66.45 ± 75.20	66.20 ± 64	< 0.001	< 0.001	< 0.001
Extension(N) Median ± IQR	77.30 ± 119.40	40.85 ± 82.30	68.90 ± 70.80	64 ± 83.60	< 0.001	< 0.001	< 0.001
Abduction(N) Median ± IQR	75.15 ± 78.80	50.95 ± 80.10	60.90 ± 72.50	60.90 ± 63.60	< 0.001	< 0.001	0.003
Adduction (N) Median ± IQR	52.20 ± 77.90	36.20 ± 72.90	55.80 ± 68.10	51.60 ± 56.50	< 0.001	< 0.001	0.001
External rotation (N) Median ± IQR	51.10 ± 54.90	34.45 ± 48.50	45.80 ± 39.10	48 ± 43.60	< 0.001	0.003	< 0.001
Internal rotation (N) Median ± IQR	56 ± 89.20	38.65 ± 60	54.95 ± 78.30	57.30 ± 71.60	< 0.001	< 0.001	< 0.001
**Non affected side**							
Supraspinatus tendon thickness (mm) ($\overline{X}$ ± SD)	4.46 ± 1.05	4.58 ± 1.07	4.68 ± 1.08	4.54 ± 1.13	0.44	0.47	0.73
AHD (mm) ($\overline{X}$ ± SD)	12.84 ± 3.12	12.62 ± 2.36	12.58 ± 2.48	12.65 ± 2.69	0.59	0.48	0.64
**Shoulder active range of motion**							
Flexion (º) Median ± IQR	180 ± 35	180 ± 60	180 ± 30	180 ± 25	> 0.999	> 0.999	> 0.999
Extension (º) Median ± IQR	70 ± 42	69 ± 40	70 ± 33	70 ± 33	> 0.999	> 0.999	> 0.999
Abduction (º) Median ± IQR	180 ± 46	180 ± 80	180 ± 35	180 ± 10	> 0.999	> 0.999	0.864
Adduction (º) Median ± IQR	35 ± 5	35 ± 5	35 ± 5	35 ± 0	> 0.999	> 0.999	> 0.999
External rotation (º) Median ± IQR	101 ± 43	108 ± 60	107 ± 46	109 ± 42	0.7245	> 0.999	> 0.999
Internal rotation (º) Median ± IQR	40.50 ± 56	40 ± 48	41.50 ± 39	40 ± 32	> 0.999	> 0.999	> 0.999
**Shoulder muscular strength**							
Flexion (N) Median ± IQR	69.10 ± 85.40	59.10 ± 75.20	68 ± 71.70	66.70 ± 63.20	0.27	0.01	0.94
Extension(N) Median ± IQR	78.45 ± 125.50	62.90 ± 87.20	75.35 ± 79.50	67.60 ± 87.20	0.04	0.05	> 0.999
Abduction(N) Median ± IQR	75.80 ± 70.80	61.80 ± 73.40	68.90 ± 85.67	63.60 ± 53.40	0.27	0.37	> 0.999
Adduction (N) Median ± IQR	66 ± 66	48.65 ± 52.70	57.10 ± 59.10	54.20 ± 45.40	0.006	0.05	0.45
External rotation(N) Median ± IQR	51.55 ± 54.30	41.75 ± 51.10	48.20 ± 76.10	45.80 ± 38.40	0.008	< 0.001	0.19
Internal rotation(N) Median ± IQR	59.60 ± 102.10	52.20 ± 54.10	57.15 ± 51.60	58.70 ± 54.70	0.03	0.19	0.02

$\overline{X}$ mean, *SD* standard deviation, *AHD* Acromiohumeral distance, *IQR* interquartile range, *N* Newtons.

Few cases of liquid in the subacromial bursa were found. On the affected side, there were three cases in the pre-surgical assessment (two of them bilateral), four in the 7–10 days after surgery (A_1_, one bilateral), five at the 3-month follow-up (A_2_), and five at the 6-month follow-up (A_3_) after surgery (one bilateral). At the 6-month follow-up (A_3_) there were two cases (6.6%) of liquid in the subacromial bursa and pain in the affected shoulder.

### Shoulder active range of motion and muscular strength

Regarding shoulder range of motion of the affected side, a decrease (p < 0.001) was observed in flexion and abduction between the pre-surgical (A_0_) and post-surgical (A_1_) assessments, and a statistically significant increase (p < 0.001) was found between post-surgical (A_1_) and 3-month (A_2_) and 6-month (A_3_) follow-up assessments. Shoulder extension increased between post-surgical (A_1_) and 3-month (A_2_) and 6-month (A_3_) follow-up assessments, and statistically significant differences were found (p < 0.001, p = 0.001) (Table 2).

Regarding muscle strength on the affected side, for all movements, a decrease (p < 0.001) between pre-surgical (A_0_) and post-surgical (A_1_) assessments and an increase (p < 0.05) between post-surgical (A_1_) and 3-month (A_2_) and 6-month (A_3_) follow-up assessments were observed. On the unaffected side, there were statistically significant increases in strength between post-surgical (A_1_) and 3-month (A_2_) follow-up assessments in flexion (p = 0.016) and external rotation (p < 0.001) and between post-surgical (A_1_) and 6-month (A_3_) follow-up assessments in internal rotation (p = 0.022). Statistically significant decreases in muscle strength were observed between pre-surgical (A_0_) and post-surgical (A_1_) assessments in extension (p = 0.0427), adduction (p = 0.006), external rotation (p = 0.008), and internal rotation (p = 0.033) (Table 2). These scores were also clinically meaningful.

### Pain, perceived shoulder disability, and health-related quality of life

**Figure 1:**
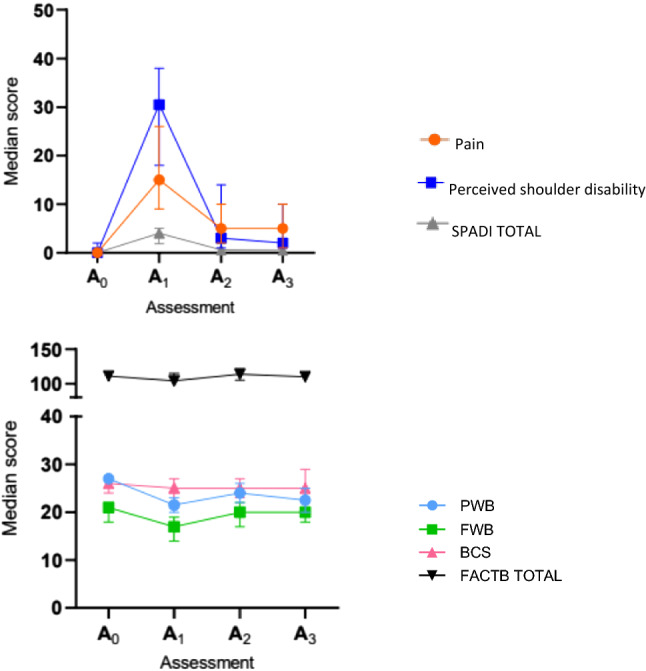
SPADI and FACT-B questionnaires score throughout follow-ups. *SPADI* Shoulder Pain and Dissability Index Questionnaire, *FACTB + 4* Functional Assesment of Cancer Therapy-Breast Questionnaire 4, *PWB* Physical Well-Being, *FWB* Functional Well-Being, *BCS* Breast Cancer Scale *A*_*0*_ pre-surgical evaluation, *A*_*1*_ post-surgical assessment, *A*_*2*_ 3 months assessment after surgery, *A*_*3*_ 6 months assessment after surgery.

The total SPADI score and the pain and disability subscale scores increased and showed statistically significant differences (p < 0.05) at the post-surgical assessment (A_1_). Then the scores decreased throughout subsequent follow-up assessments (Table 3, Fig. 1). Regarding HRQoL, the PWB and FWB subscale scores decreased (p < 0.05) between the pre-surgical (A_0_) and post-surgical (A_1_) assessments. The FWB subscale score improved at the 3-month follow-up (A_2_) (Table 3, Fig. 1).

**Table 3:** Perceived shoulder pain and disability and health related quality of life between baseline measurement and different follow-ups.

Outcomes	Assessments				P-values		
	Pre-surgical A_0_	7–10 days post-surgical A_1_	3- month post-surgical A_2_	6- month post-surgical A_3_	A_0_ vs A_1_	A_1_ vs A_2_	A_1_ vs A_3_
SPADI TOTAL Median ± IQR	0 ± 3.69	4.03 ± 7.76	0.61 ± 5.07	0.53 ± 8.53	< 0.001	< 0.001	< 0.001
Pain Scale Median ± IQR	0 ± 21	15 ± 65	5 ± 44	5 ± 46	< 0.001	0.05	0.001
Disability Scale Median ± IQR	0 ± 27	30.5 ± 60	3 ± 31	2 ± 65	< 0.001	< 0.001	< 0.001
FACT-B Median ± IQR	110.75 ± 64	104 ± 47	114 ± 73	110 ± 74	0.80	0.09	> 0.99
PWB Median ± IQR	27 ± 17	21.5 ± 14	24 ± 17	22.5 ± 15	< 0.001	0.30	> 0.99
FWB Median ± IQR	21 ± 16	17 ± 18	20 ± 17	20 ± 20	0.03	0.01	0.38
BCS Median ± IQR	26 ± 18	25 ± 16	25 ± 23	25 ± 23	0.48	> 0.99	> 0.99

*VAS* Visual Analog Scale, $\overline{X}$ mean, *SD* standard deviation, *IQR* interquartile range, *PWB* physical well being, *FWB* functional well being, *BCS* breast cancer scale.

A Pearson correlation coefficient of 0.639 was found only at the 3-month follow-up for the association between the SPADI and FACTB + 4 scores.

## Discussion

To the best of the authors’ knowledge, no other studies have analyzed shoulder US findings in women treated for breast cancer with pre-surgical and post-surgical follow-up over a 6-month period. In this longitudinal study we did not use matched controls due to the potential drawbacks, including extra cost, added time for enrollment, potential dropouts in follow-up, and increased bias.

Our results suggest that there is decreased thickness of the supraspinatus tendon 6 months after surgery, decreased active range of motion in the affected shoulder, changes in muscular strength in both shoulders, increased shoulder pain intensity, and declined quality of life after surgery.

### Supraspinatus tendon thickness, acromiohumeral distance, and the presence of fluid in the subacromial bursa

The decreased supraspinatus tendon thickness could be related to the appearance of a symptomatic or asymptomatic disease of the rotator cuff, which is considered a common age-related pathology^6,29,30^. Moreover, previous studies reported that a reduction in tendon thickness may be caused by creep (i.e., alignment of collagen fibers in the direction of applied stress) or fluid transfer out of the tendon^31,32^. Therefore, intrinsic factors such as age and possible muscle weakness as well as extrinsic factors from surgery could contribute to the reduced tendon thickness. The mean age of the women included in this study agrees with previous research showing a high prevalence of shoulder pathology related to the rotator cuff above 60 years of age^29,30^. Therefore, linking only supraspinatus tendon deterioration with medical treatment for breast cancer could be misleading; although statistically significant differences were found, the effect size is small and the change did not reach the MCD at 6 months.

It would be interesting to extend the follow-up period, because structural tendon changes can take a long time to detect, as reflected in studies that examined asymptomatic rotator cuff tears with annual ultrasound assessments for a follow-up period of at least 2 years^33,34^.

Two studies reporting on US assessment of the supraspinatus tendon in women with breast cancer^35,36^ were found. Although both studies agree with ours regarding the lack of correlation between US findings and symptoms or physical examinations, they analyzed different samples and had methodological differences that prevent a comparison. Both were cross-sectional studies, with follow-up of 38 women with lymphedema^35^ and 52 women with chronic shoulder pain^36^ at 1.5 and 4 years after breast cancer surgery, respectively. In addition, neither study used the same exploration methodology for ultrasound measurements. Both studies reported a high prevalence of long-term structural alterations of the supraspinatus muscle tendon in women with breast cancer, consistent with the results of Lauridsen et al.^37^, and hypothesized that structural changes were the possible cause of shoulder pain in these women.

Medium- and long-term follow-up could prevent future complications, as suggested by a systematic review and meta-analysis in which chronic asymptomatic changes that can occur in tendon morphology and observed with US were shown to be predictive of subsequent development of tendinopathy^38^. Another study found that approximately 25–50% of asymptomatic rotator cuff tears will become symptomatic within 2–3 years^34^. Therefore, this population could be considered at possible risk of developing symptoms related to rotator cuff disease in the future. On the other hand, rotator cuff disease is multifactorial^39^ and can involve more complex entities, such as pain, biopsychosocial factors, mechanical overload, etc. Knowing that these morphological tendon alterations could occur makes it possible to foresee morphological and symptomatic worsening and include strategies aimed at improvement.

No statistically significant differences were found in the AHD between the affected and unaffected sides or throughout the 6 months after surgery. Two previous studies^21,40^ found no differences in AHD between symptomatic patients with subacromial syndrome and asymptomatic subjects; however, two other studies^41,42^ did find differences. Our results agree with the former, more recent studies^21,40^.

### Shoulder active range of motion and muscular strength

Our results suggest that statistically significant and minimal detectable change in active range of motion mainly occur in the shoulder on the affected side, especially in flexion and abduction movements. Many studies have reported similar results in women treated for breast cancer^1,2,43,44^, and in women who did not have upper limb pain^3^, which was the case for most of the women in this study.

There was a statistically significant and clinically meaningful decrease in muscle strength in all movements on the affected side between the pre-surgical and post-surgical assessments, and it was restored throughout subsequent follow-ups. The lowest strength was at the post-surgical assessment (between 7 and 10 days later), when the women did not move their arms over long distances, exert effort, carry weights, etc., which is in accordance with medical guidelines and the presence of pain causing inhibition of muscle activity^13^; thus, these results were predictable. Statistically significant changes in strength were also observed on the unaffected side. As with the affected side, these changes were also clinically meaningful at the post-surgical assessment. Many studies have reported similar decreases in shoulder strength in women treated for breast cancer^1,2,43,44^.

Regarding the active range of motion and strength of the shoulder, a recent study reported that women treated for breast cancer showed alterations in shoulder neuromuscular activity, in both the onset and amplitude of muscle activity, and the changes seemed to be more significant in the presence of persistent pain^45^. These alterations have also been observed in subacromial syndrome as a possible compensation for rotator cuff deficiencies^46,47^.

The most current neuroscience studies suggest that the initial peripheral sensitization caused by surgery and medical treatment for breast cancer can inhibit the primary motor cortex by altering the motor strategy of the shoulder, overloading peripheral structures, and triggering more nociceptive input^13,14^. In addition, shoulder alterations such as decreased range of motion and loss of strength in the external rotators have been observed to persist for up to 5 years after medical and/or surgical treatment^48^, so peripheral structures may continue to be overloaded in the long term.

### Pain, perceived shoulder disability, and health-related quality of life

The pain intensity score was not high, but it should not be ignored, since the number of women with shoulder pain progressively increased after the post-surgical assessment. Most studies^1,2,10,43^ reported a prevalence of persistent shoulder pain between 20 and 47% 1–3 years after surgery, so although the data are not comparable, they point to the same trend.

The total score and the pain and disability subscale scores on the SPADI questionnaire were worse at the post-surgical assessment but progressively recovered over time, with no significant differences between pre-surgical assessment and 6-month follow-up. Worsening pain and disability of the affected shoulder after axillary and breast surgery and/or medical treatment due to initial peripheral sensitization are considered clinically normal^13^.

Significant differences in quality of life were only detected on the PWB and FWB subscales between pre-surgical and 6-month follow-up, and only the change in the PWB dimension persisted as the main sequela, in agreement with a systematic review performed in 2017^44^.

### Study limitations

A larger sample size would have allowed a comparison of results by axillary surgery groups. A longer follow-up would have allowed an observation of the thickness of the supraspinatus tendon over a longer period of time. Data related to medications and sports practices were not collected, therefore it is unknown whether they might have influenced the results. Complementing the US protocol with a dynamic US assessment would have provided interesting information on the behavior of moving structures^49^.

### Practice implications

Ultrasound can help physiotherapists to detect early objective changes in the tendon in order to initiate preventive treatment. It would be interesting to include US assessment for women undergoing long-term medical treatment for breast cancer, especially those who suffer from shoulder dysfunction and/or persistent pain.

## Conclusions

A trend of decreasing thickness of the tendon of the supraspinatus muscle of the shoulder on the affected side was observed in women treated for breast cancer. This structural change, which was observed early by ultrasound, could contribute to shoulder dysfunction in breast cancer survivors. A decrease in active range of motion of the shoulder on the affected side after surgery was observed, and this persisted for 6 months after surgery in 10% of women. Physical and functional well-being decreased after surgery, and decreased physical well-being persisted for 6 months after surgery.

Further studies with a follow-up of at least 12 months after medical treatment for breast cancer with a larger sample size to evaluate ultrasound findings of the shoulder are needed.

## Supplementary Information

Supplementary Information.

## Data Availability

Data are held securely by the research team and may be available upon reasonable request and with relevant approvals in place.
