# Supplementary material for: Changes in shoulder outcomes using ultrasonographic assessment of breast cancer survivors: a prospective longitudinal study with 6-month follow-up
Source: Sci Rep. 2021 Nov 26;11:23016. doi: 10.1038/s41598-021-02379-9 (PMC8626448; doi:10.1038/s41598-021-02379-9)
Supplement: Supplementary file 1 — Supplementary Information. [file 41598_2021_2379_MOESM1_ESM.docx]

**SUPPLEMENTARY FILE. ULTRASONOGRAPHIC ASSESSMENT PROTOCOL**

1. Thickness of the supraspinatus tendon (21): The participants were seated, with their feet flat on the floor, a neutral trunk posture and their heads facing forward. The subject' s upper limb was placed in the modified Crass position (Figure S1). The ultrasound transducer was placed in the anterior aspect of the shoulder in short axis, perpendicular to the supraspinatus tendon and just anterior to the antero-lateral margin of the acromion to capture the supraspinatus tendon and the long head of the biceps tendon laterally. We took measurements between the echogenic limits of the tendon at points 5 mm and 10 mm from the posterior border of the biceps tendon. The tendon delimitation was defined inferiorly as the first hyperechoic region on the anechoic articular cartilage of the head of the humerus and the hyperechoic superior border of the tendon below the subdeltoid bursa (Figure S1).


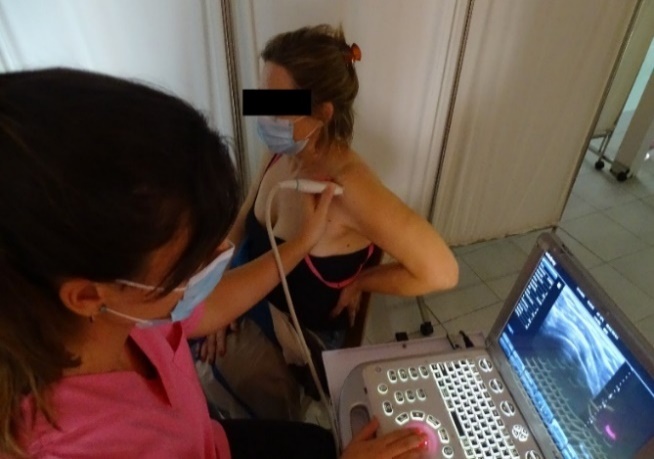

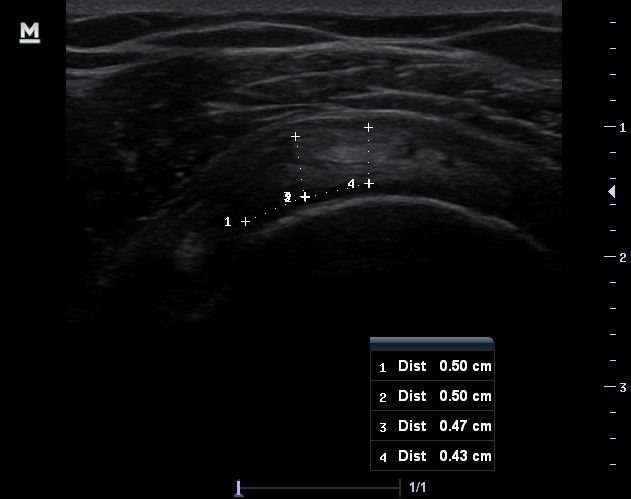


**Figure S1.** Left: Woman in the modified Crass position and placement of the ultrasound transducer for the evaluation of the thickness of the supraspinatus tendon. Right: Ultrasound image and measurement of the thickness of the supraspinatus tendon.

1. Acromiohumeral distance (21,22): The subject seated with the feet resting on the floor, a neutral trunk posture with the head facing forward and the upper limb at rest with the elbow flexed at 90º and hands resting on the lap. The transducer was placed in the anterior aspect of the anterior margin of the acromion, conﬁrmed by palpation, with the long axis of the transducer positioned in the plane of the scapula and parallel to the surface of the acromion (Figure S2). Once both the acromion and humerus were visualized, the transducer was moved forward until the most anterior part of the acromion is in view, with a clear view of the humeral head below, at which point the image was captured. The acriomiohumeral distance (AHD) was measured as the linear distance between the superior aspect of the humeral head and the inferior aspect of the acromion (Figure S2).


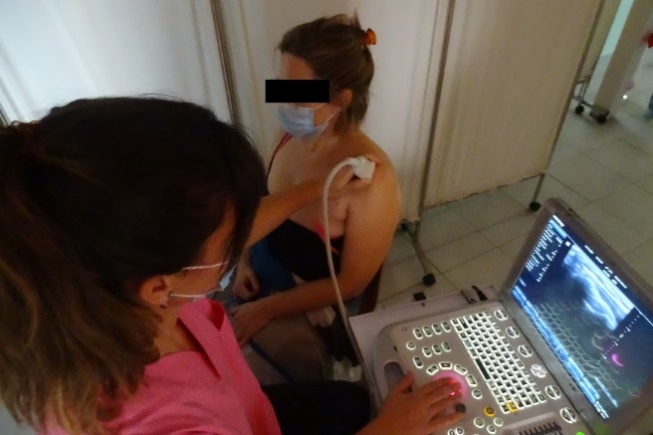

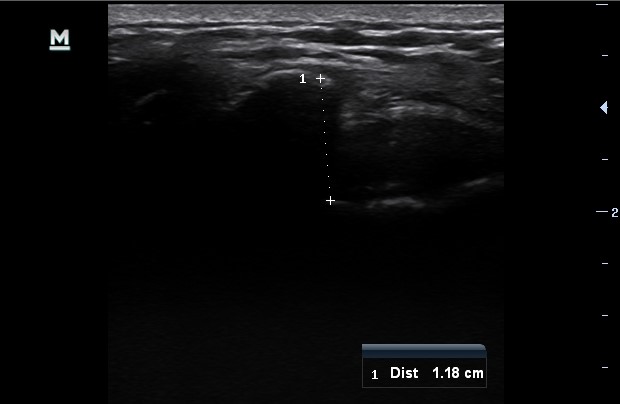


**Figure S2**. Left: Position of the woman and the ultrasound transducer for the measurement of the acromiohumeral distance. Right: Ultrasound imaging and measurement of acromiohumeral distance.
